# Targeting the Multiple Physiologic Roles of VDAC With Steroids and Hydrophobic Drugs

**DOI:** 10.3389/fphys.2020.00446

**Published:** 2020-05-07

**Authors:** Tatiana K. Rostovtseva, María Queralt-Martín, William M. Rosencrans, Sergey M. Bezrukov

**Affiliations:** Section on Molecular Transport, Eunice Kennedy Shriver National Institute of Child Health and Human Development, National Institutes of Health, Bethesda, MD, United States

**Keywords:** mitochondrial outer membrane, voltage dependent anion channel, planar membrane, alpha-synuclein, tubulin, pharmacology, gramicidin A

## Abstract

There is accumulating evidence that endogenous steroids and non-polar drugs are involved in the regulation of mitochondrial physiology. Many of these hydrophobic compounds interact with the Voltage Dependent Anion Channel (VDAC). This major metabolite channel in the mitochondrial outer membrane (MOM) regulates the exchange of ions and water-soluble metabolites, such as ATP and ADP, across the MOM, thus governing mitochondrial respiration. Proteomics and biochemical approaches together with molecular dynamics simulations have identified an impressively large number of non-polar compounds, including endogenous, able to bind to VDAC. These findings have sparked speculation that both natural steroids and synthetic hydrophobic drugs regulate mitochondrial physiology by directly affecting VDAC ion channel properties and modulating its metabolite permeability. Here we evaluate recent studies investigating the effect of identified VDAC-binding natural steroids and non-polar drugs on VDAC channel functioning. We argue that while many compounds are found to bind to the VDAC protein, they do not necessarily affect its channel functions *in vitro*. However, they may modify other aspects of VDAC physiology such as interaction with its cytosolic partner proteins or complex formation with other mitochondrial membrane proteins, thus altering mitochondrial function.

## Introduction

The role of mitochondria in oxidative metabolism, apoptosis, and steroidogenesis is well established and mitochondrial malfunction plays a central role in a broad range of disorders. Not surprisingly, pharmacological targeting of mitochondria has the potential to provide therapeutic benefits in a wide range of diseases related to mitochondrial dysfunction, including cancer, obesity, cardiovascular, and neurodegenerative diseases (such as Amyotrophic Lateral Sclerosis, Alzheimer’s, Huntington’s, and Parkinson’s) (Lin and Beal, 2006a, b; D’Souza et al., 2011; Guo et al., 2016; de Mello et al., 2018; Bonora et al., 2019; Grunewald et al., 2019). Despite its central role in a number of diseases, currently there are no approved drugs that directly target mitochondria (Wang et al., 2016).

**FIGURE 1:**
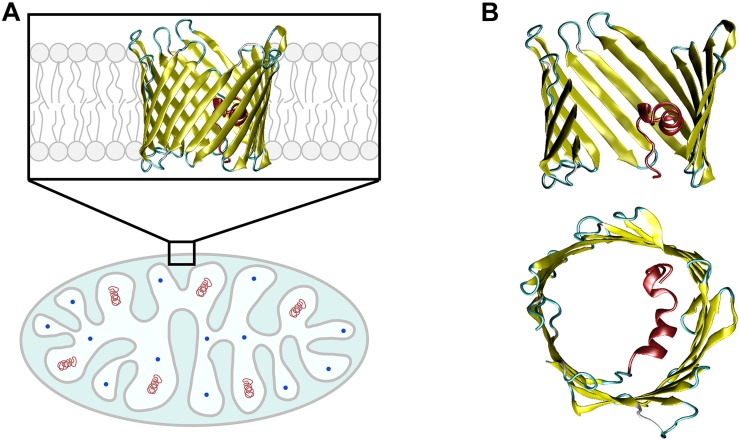
VDAC is the major transport channel of the mitochondrial outer membrane. **(A)** The mitochondrion is a double-membrane organelle with inner (IM) and outer (MOM) membranes, with VDAC embedded in the MOM. **(B)** Side and top views of mouse VDAC1 isoform (mVDAC1, PDB ID: 3EMN). In the side view, part of the β-barrel is cropped to show the N-terminal α-helix (in red). Original figure.

The mitochondrial outer membrane (MOM) is the interface between mitochondria and cytosol, acting as a “mediator” between the energy-producing mitochondrion and the rest of the cell. The MOM controls normal metabolite and energy exchange between mitochondria and the cytoplasm, and its permeabilization triggers cell death by releasing apoptogenic factors into the cytosol. Thus, tight regulation of MOM permeability is crucial for cell metabolism, and its alteration is often associated with mitochondrial dysfunction. A significant portion of MOM permeability is controlled by the voltage-dependent anion channel (VDAC), the major metabolite channel and most abundant protein in the MOM (Figure 1A; Colombini, 2004; Lemasters and Holmuhamedov, 2006). VDAC constitutes the main pathway for the exchange of small ions, ATP, ADP, and other water-soluble mitochondrial metabolites across the MOM and serves as a conjunction point for a variety of cell signals through its association with numerous cytosolic proteins (Colombini, 2004; Lemasters and Holmuhamedov, 2006; Rostovtseva and Bezrukov, 2008), thus serving as a global regulator of mitochondrial energetics and cell metabolism.

Due to its central role in controlling MOM permeability, and consequently mitochondrial function, VDAC is emerging as a promising pharmacological target for treating a wide variety of mitochondria-associated pathologies (Shoshan-Barmatz and Ben-Hail, 2012; Camara et al., 2017; Karachitos et al., 2017; Shoshan-Barmatz et al., 2018b). In this context, proteomics and biochemical approaches along with molecular dynamics simulations have identified a large number of non-polar compounds, such as natural and synthetic steroids, small-molecule anticancer and neuroprotective drugs, and general anesthetics, which are able to bind to VDAC (Shoshan-Barmatz and Ben-Hail, 2012; Magri and Messina, 2017; Reina and De Pinto, 2017; Leanza et al., 2019). These findings have sparked speculation that both natural steroids and synthetic hydrophobic drugs regulate mitochondrial physiology by directly affecting VDAC *channel* properties, despite the lack of experimental evidence in most of the cases.

In this paper, we review previous studies identifying VDAC-binding natural steroids and non-polar drugs and discuss their effect on VDAC channel function *in vitro*. We show that while some hydrophobic compounds bind to VDAC and *affect* its channel properties *in vitro*, others directly interact with the protein but *do not alter* channel function. We discuss the available experimental strategies to probe the effect of hydrophobic compounds on VDAC channel functional properties and consequently, the related obstacles in interpreting results.

We espouse the scenario that non-polar compounds targeting VDAC do not necessarily affect its basic channel properties such as permeability, selectivity, and voltage-gating. Instead, they may interfere with VDAC interaction with its cytosolic partners, such as tubulin, α-synuclein, hexokinase, and Bcl-2 family proteins, or with other mitochondrial membrane proteins, such as translocator protein 18 kDa (TSPO).

Considering the established unique role of VDAC in regulation of mitochondrial physiology (600 vs 9,000 usages in Google Scholar) (Colombini, 2004; Lemasters and Holmuhamedov, 2006; Shoshan-Barmatz et al., 2018a), the identification of pharmacological agents for VDAC regulation is absolutely crucial for improving outcomes of mitochondria-associated diseases (Camara et al., 2017; Magri and Messina, 2017; Reina and De Pinto, 2017). There is an urgent need for a deeper knowledge about the molecular mechanisms of VDAC interaction with plethora of structurally and functionally unrelated endogenous proteins and steroids and synthetic drugs. Here we discuss the strategies to rationalize the mechanisms of action of natural steroids, small molecule drugs, and synthetic compounds targeting VDAC.

## Overview of VDAC Basic Channel Properties

VDAC has three isoforms in mammals, VDAC1, VDAC2, and VDAC3, with varied distribution *in vivo* depending on species and tissue types (Messina et al., 2012). In general, VDAC1 and VDAC2 have higher expression levels (∼90%) than the less abundant VDAC3 (∼10%), with exception for testis (Rahmani et al., 1998; Messina et al., 2012). Although a common transport function may be attributed to all VDAC isoforms, there appear to be unique physiological roles that are not well understood. While VDAC1 knockout causes only mild mice growth retardation and defects in mitochondrial respiration (Anflous et al., 2001), VDAC2 is the only isoform whose knockout is essentially lethal in mice embryos and it is required for BAX/BAK-induced apoptosis (Cheng et al., 2003; Chin et al., 2018). VDAC3-knockout mice show a peculiar phenotype – male infertility – not observed with VDAC1 deficiency (Sampson et al., 2001). The vast majority of studies refer to VDAC1, the most abundant VDAC isoform in the MOM of most cells (Homble et al., 2012; Messina et al., 2012) and the only mammalian isoform with a resolved 3D structure (Bayrhuber et al., 2008; Hiller et al., 2008; Ujwal et al., 2008). VDAC is an integral membrane protein that, in its native lipid membrane environment, adopts a β-barrel conformation with 19 β-strands and an α-helical N-terminus located approximately in the middle of the pore (Figure 1B; Bayrhuber et al., 2008; Hiller et al., 2008; Ujwal et al., 2008). Although there are no current structures of mammalian VDAC2 and VDAC3, the high degree of sequence similarity enables the construction of reliable homology models showing a conserved 3D structure (Amodeo et al., 2014). All three VDAC isoforms form almost identical anion-selective and voltage-gated ion channels *in vitro* when reconstituted into planar lipid membranes (Maurya and Mahalakshmi, 2015; Queralt-Martin et al., 2020).

**FIGURE 2:**
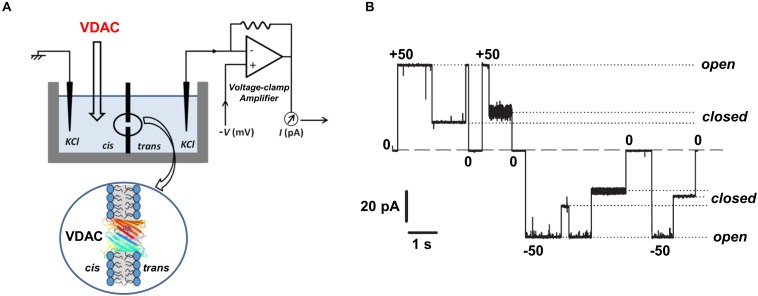
Experimental assessment of VDAC channel properties. **(A)** A schematic experimental setup for VDAC reconstitution showing a planar lipid membrane chamber consisting of two compartments, *cis* and *trans*, and the model circuit for current amplification and registration. The cartoon below represents a planar lipid bilayer with a reconstituted VDAC channel. **(B)** A representative single-channel current trace obtained with VDAC reconstituted into a lipid membrane; membrane-bathing solution consists of 250 mM KCl buffered with 5 mM HEPES at pH 7.0. Under high applied voltages (± 50 mV) channel conductance moves from a high conducting “open” state to various low conducting “closed” states. Relaxing the voltage to 0 mV reopens the channel. Dashed lines indicate the zero current level and dotted lines indicate open and closed states. Adapted by permission from Rostovtseva and Bezrukov (2015). Copyright © Springer International Publishing Switzerland 2015.

*In vitro* ion channel reconstitution of isolated membrane protein is, so far, the best available method to directly study the functional properties of organelle membrane channels like VDAC. A schematic of the experimental setup typically used for VDAC channels reconstitution and monitoring their currents is shown in Figure 2A. When reconstituted in planar lipid bilayers, VDAC forms large conducting channels (4 nS in 1 M or 0.7 nS in 150 mM KCl) with a mild preference for anions over cations: the ratio of Cl^–^/K^+^ permeabilities is between 1.5 and 2.3, depending on salt concentration in the membrane-bathing solutions (Zambrowicz and Colombini, 1993; Colombini et al., 1996; Krammer et al., 2014; Queralt-Martin et al., 2020). VDAC channels are permeable to negatively charged metabolites and non-charged polymers with molecular weight up to 2 kDa (Colombini, 1980; Gurnev et al., 2011). Under applied voltages (typically exceeding 30 mV), VDAC reconstituted in planar membranes transitions from a unique high-conductance, “open” state to a variety of low-conductance, so-called “closed” states, of ∼0.5 of the open state conductance (Figure 2B). Once the transition to the closed states occurs, the channel can remain in them for a prolonged time, depending on the amplitude of the applied voltage. Often, reduction of the voltage to 0 mV is required to reopen the channel (Figure 2B). Importantly, closed states of VDAC are virtually impermeable to negatively charged metabolites such as ATP due to the steric and electrostatic barriers which prevent ATP translocation (Rostovtseva and Colombini, 1996; Rostovtseva and Colombini, 1997; Noskov et al., 2013). The transitions between different conductance states under applied voltage, or *voltage-gating*, is a characteristic and conserved property of VDAC channels reconstituted into lipid membranes and its namesake. A question about whether voltage gating occurs in live cells or if this is a peculiarity of *in vitro* systems remains to be answered (for the related discussion; see Colombini, 2004; Rostovtseva and Bezrukov, 2008). The magnitude and nature of the MOM transmembrane potential is still a matter of debate. The main source of this potential is believed to be the so-called “Donnan potential” which arises from high concentration of VDAC-impermeable charged macromolecules at both sides of the MOM and ranges from 10 to up to 40 mV according to some estimates (Porcelli et al., 2005; Lemeshko, 2006). VDAC in complex with hexokinase was recently suggested as another source of the MOM potential, estimated to be as high as 50 mV (Lemeshko, 2014a, b). Moreover, VDAC gating *in vivo* could be facilitated by substantial osmotic effects arising from the crowded environment of the mitochondrial intermembrane space and cytosol (Zimmerberg and Parsegian, 1986). In comparison with the single channel conductance and ion selectivity, the two basic ion channel properties, VDAC voltage gating is the most sensitive to modulation by environmental factors, such as lipid membrane composition, medium pH, salt concentration, and osmotic stress (Bowen et al., 1985; Zimmerberg and Parsegian, 1986; Colombini, 1989; Rostovtseva et al., 2006; Teijido et al., 2014; Mlayeh et al., 2017; Queralt-Martin et al., 2019). The mechanism of VDAC gating, as well as that of other β-barrel channels (like bacterial porins or toxin channels), is still enigmatic, and all available structural data on VDAC relate so far to its open conformation. The lack of a resolved structure for the closed-state conformation and an unclear mechanism of its closure make a prediction of the effect of a specific compound on VDAC gating using MD simulations rather challenging, with a risk of ambiguous interpretation.

**FIGURE 3:**
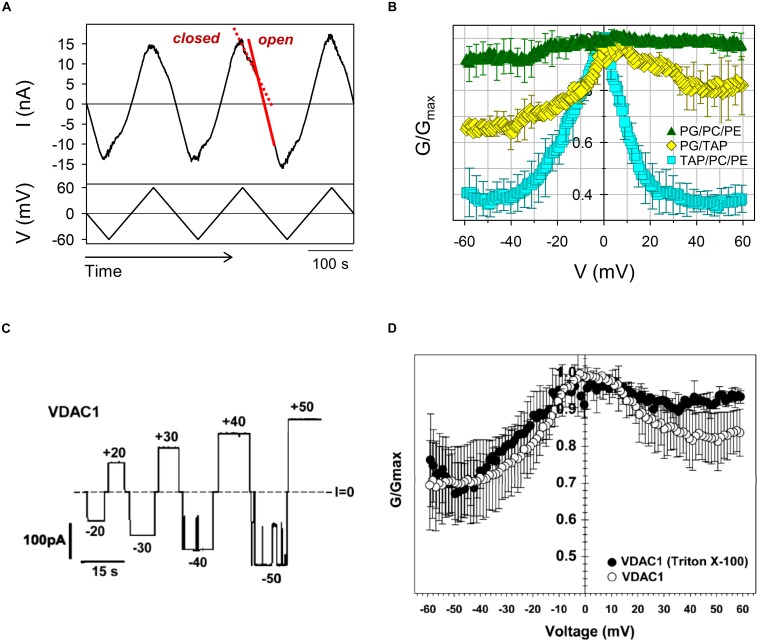
Asymmetry of VDAC gating in respect to voltage polarity. **(A)** A representative trace of ion current through multiple VDAC channels (upper panel) in response to a triangular voltage wave (5 mHz, ± 60 mV, bottom panel) employed to evaluate VDAC gating. Steep slopes at low potentials correspond to the high conductance of open channels (red solid line), whereas the reduced irregular slopes at higher potentials correspond to the lower conductance of closed states (red dashed line). **(B)** VDAC gating decreases with negative lipid content of the membranes. The normalized conductance, *G/G_*max*_*, of mouse VDAC1 as a function of the applied voltage obtained on multichannel membranes formed from anionic (DOPG/DOPC/DOPE 2:1:1), cationic (DOTAP/DOPC/DOPE 2:1:1), or neutral (DOPG/DOTAP 1:1) lipid mixtures. *G* is the conductance at a given voltage and *G*_*max*_ is the maximum conductance at | V| ≤ 10 mV. The membrane-bathing solutions consisted of 150 mM KCl buffered with 5 mM HEPES at pH 7.4. Adapted from Queralt-Martin et al. (2019). Copyright (2019), with permission from Elsevier. **(C)** A single-channel record of the human VDAC1 reconstituted into a neutral DPhPC membrane at different applied voltages as indicated. **(D)** Characteristic bell-shape plots of the normalized average conductance vs. the applied voltage. The membrane-bathing solutions consisted of 1 M KCl buffered with 5 mM HEPES at pH 7.4. **(C,D)** Were adapted with permission from Eddy et al. (2012). Copyright (2012) American Chemical Society.

VDAC gating is a stochastic process (Pustovoit et al., 2006; Banerjee, 2015; Rappaport et al., 2015), where the exact timing of a gating event cannot be predicted. Therefore, a consistent quantification of gating must rely on strong statistics. Single-channel experiments usually provide limited statistics due to the difficulty in obtaining the high number of necessary repeats: at least ∼100 events are required for a reliable fitting of the exponential distribution of times obtained at a given applied voltage (Nestorovich and Bezrukov, 2004; Perini et al., 2019). Alternatively, enough gating events can be collected following the experimental protocol developed by Colombini and coworkers (Schein et al., 1976; Colombini, 1989; Rostovtseva et al., 2006), in which a slow voltage triangular wave is applied to multiple VDAC channels inserted in the planar lipid membrane (Figure 3A). The essence of this method is based on the observation that, contrary to the relatively slow process (with a characteristic time on the order of tens of seconds) of channel closure at high voltages, the channel reopening at the decreased voltages is fast (on the order of milliseconds) and could be used to study an equilibrium-like behavior. If the rate of voltage variation – the frequency of the periodic triangular wave of the voltage – is low enough, it ensures equilibration of the channel reopening. Accordingly, channel reopening can be approximated as a quasi-equilibrium two state Markovian process (Rappaport et al., 2015) and is satisfactorily described by the Boltzmann equation. Following this formalism, gating is usually characterized by two parameters: the gating charge, *n*, and the characteristic voltage of equipartitioning where half the channels are open and half are closed, *V*_0_.

**FIGURE 4:**
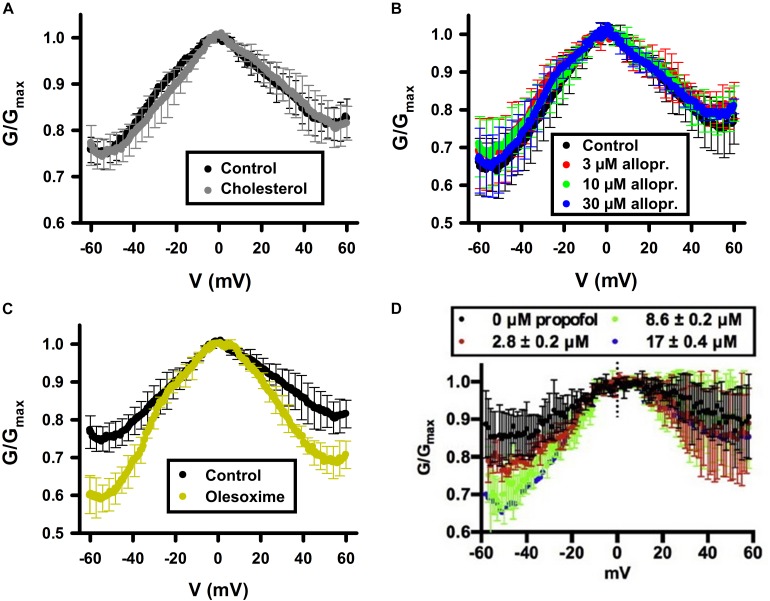
Cholesterol and allopregnanolone do not affect VDAC voltage gating, while olesoxime and propofol do. Normalized conductance as a function of the applied voltage obtained with multichannel VDAC membranes formed from PLE in the absence (control) or presence of 4% (w/w) of cholesterol **(A)**; from pure PLE **(B)**; or in the absence (control) or presence of 16% (w/w) of olesoxime in PLE/cholesterol (4% w/w and 16% w/w, respectively) membrane **(C)**. Allopregnanolone **(B)** and propofol **(D)** were added to the membrane-bathing solutions of 1M KCl buffered by 5 mM HEPES at pH 7.4 in final concentrations indicated. In **(D)** membranes were formed from DOPC:DOPE (1:1) mixture. In **(A,C,D)**, VDAC was purified from rat liver mitochondria and recombinant mVDAC1 was used in **(B)**. **(A,C)** Were adapted by permission from Springer Nature: Springer International Publishing Cellular and Molecular Life Sciences (Rovini et al., 2019), COPYRIGHT (2019); **(B)** was adapted from Cheng et al. (2019), Copyright (2019), with permission from Elsevier; **(D)** was adapted from Weiser et al. (2014a) with permission from John Wiley and Sons.

Current during channel reopening is analyzed to build conductance vs. voltage (*G/V*) plots. To take into account a variable number of channels in each experiment (channel insertion is also a spontaneous process, and the number could vary significantly even during one experiment), the *G/V* plots are expressed as a normalized conductance (Figures 3B,D, 4) and the channel open probability is calculated from the reopening *G/V* plots, providing an adequate quantification with relevant parameters that describe the gating process (Colombini, 1989; Rostovtseva et al., 2006). Under normal pH conditions, the gating charge is about 3 and *V*_0_ varies between 25 and 35 mV (Bowen et al., 1985; Colombini, 1989; Thomas et al., 1993; Teijido et al., 2014; Queralt-Martin et al., 2019).

Another peculiar feature of VDAC gating is an asymmetry of *G/V* plots in respect to the sign of the applied voltage. This asymmetry can be seen in *G/V* plots as different relative conductances of the closed states *G*_*min*_ for different signs of the applied voltage (Figures 3B,D). This asymmetry is also observed in single channel experiments: often transitions to the closed states start at ∼10 mV lower amplitudes of applied voltages at negative polarities than at positive (Figure 3C). The gating asymmetry reflects an intrinsic property of the channel, when, depending on the voltage polarity, VDAC gates by two different processes (Colombini et al., 1996; Song et al., 1998). The absence of structural data on VDAC closed states makes further speculations unjustified. The gating asymmetry depends on lipid composition: gating is almost symmetrical in the membranes made of dioleoyl-phosphatidylcholine (DOPC) (Rostovtseva et al., 2006) or of soybean Polar Lipid Extract (PLE) (Cheng et al., 2019; Rovini et al., 2019) (Figures 4A–C), while it is markedly asymmetrical in diphytanoyl-phosphatidylcholine (DPhPC) (Eddy et al., 2012; Figure 3D) or dioleoyl-phosphatidylethanolamine (DOPE) membranes (Rostovtseva et al., 2006). It was demonstrated that the gating asymmetry is enhanced in the presence of non-lamellar lipids, such as DOPE or cardiolipin – they promote channel closure at the negative voltage polarity (Rostovtseva et al., 2006). This was interpreted as indicative of a sensitivity of the VDAC β-barrel to the lipid packing stress (Rostovtseva and Bezrukov, 2008). Given that the difference in lateral pressure of PC and PE membranes can reach up to hundreds of atmospheres (Bezrukov, 2000), the non-lamellar lipids could shift VDAC conformational equilibrium promoting transition to the low conducting states (Rostovtseva et al., 2006; Rostovtseva and Bezrukov, 2008). A later study (Mlayeh et al., 2017) suggested that PE-induced gating asymmetry in plant VDAC could be caused by direct interactions between PE headgroups and specific residues located in the VDAC loops which connect β-strands and are exposed at the membrane surface. Lipid headgroup charge modulates VDAC gating as well. Positively charged synthetic lipid dioleoyl-trimethylammonium-propane (DOTAP) enhances VDAC closure, while negatively charged dioleoyl-phosphatidylglycerol (DOPG) decreases it, compared to neutral lipid mixtures (DOPG/DOTAP) (Figure 3B; Queralt-Martin et al., 2019). In neutral DPhPC (Eddy et al., 2012; Teijido et al., 2014) or DOPC lipids (Rostovtseva et al., 2006), VDAC shows qualitatively similar gating as in 1:1 mixture of positive DOTAP and negative DOPG lipids (Queralt-Martin et al., 2019), with a noticeable difference in asymmetry depending on particular lipid composition (compare Figures 3B,D, 4). Sensitivity to phospholipid headgroup charge could arise from specific protein-lipid interactions, as was suggested for the PE headgroups (Mlayeh et al., 2017) and/or from the coupling between the mechanical pressure in the hydrocarbon lipid region and channel gating (Rostovtseva and Bezrukov, 2008).

## Choosing Strategies to Assess Effects of Drugs on VDAC Channel Properties

**FIGURE 5:**
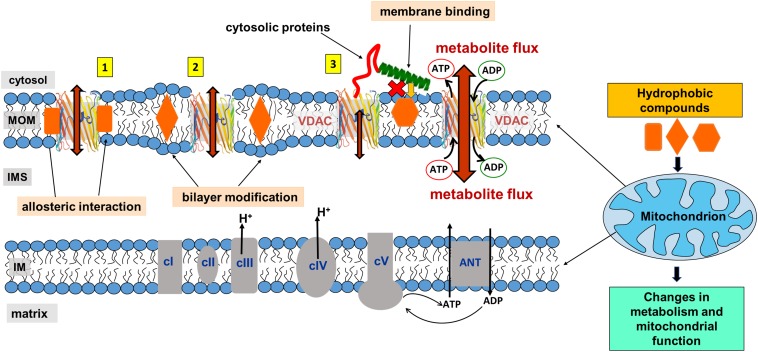
Proposed model of mitochondrial metabolism regulation by non-polar compounds through interaction with VDAC. A non-polar compound can affect VDAC channel properties either by interacting directly with the channel at the protein-lipid interface (interaction type 1), indirectly by modifying properties of the lipid bilayer surrounding VDAC (type 2), or by interfering with cytosolic regulators of VDAC at the membrane interface (type 3). Indeed, for a particular compound, a combination of all three types of interactions is also feasible. Adapted by permission from Springer Nature: Springer International Publishing Cellular and Molecular Life Sciences (Rovini et al., 2019), Copyright (2019).

When discussing the effects of non-polar compounds on VDAC gating, a problem arises from the fact that gating is sensitive to the properties of the lipid bilayer, as discussed above (Figure 3B; Rostovtseva et al., 2006; Mlayeh et al., 2017; Queralt-Martin et al., 2019). It is usually assumed that if a non-polar compound affects VDAC gating, it binds to VDAC; however, if compound partitioning to the planar membrane modifies bilayer properties by changing its hydrophobic thickness, lipid packing stress, or fluidity, then it may influence VDAC gating indirectly, through a so-called “*lipid matrix effect.*” Many hydrophobic compounds are known to change lipid membrane properties. Thus, the compound of interest could affect VDAC gating by directly interacting with the protein or indirectly, by altering the lipid bilayer surrounding the channel (a schematic in Figure 5 illustrates these two scenarios, types 1 and 2). In the latter case, the compound could also affect the function of any MOM proteins, leading to off-target effects.

Therefore, in this review for the sake of simplicity, we separate these two effects induced by the membrane-partitioning non-polar compounds as (i) an interference with lipid-protein interfacial interactions due to direct binding to the VDAC β-barrel hydrophobic external surface and (ii) a disturbance of the acyl chain packing induced by non-polar compound partitioning to the either region of lipid bilayer which could indirectly affect VDAC gating properties without direct binding to protein, the “*lipid matrix effect.*”

An alternative scenario is the lack of effect of non-polar compounds on VDAC gating, when it is often assumed that there is no interaction between the compound and the channel. However, there are several experimental and interpretational issues which must be taken into account when analyzing a system of channels reconstituted in planar lipid bilayers. Indeed, hydrophobic compounds may have limited partitioning to the lipid membrane, preferring to stay in the detergent mixed micelles in the membrane-bathing buffer solution instead. Therefore, the absence of the effect of a non-polar drug on reconstituted VDAC could be due to its poor partitioning into the lipid bilayer. A simple experimental solution to avoid this ambiguity is to add the hydrophobic compound directly to the membrane-forming lipid solution. This protocol would ensure the presence of the compound in the membrane at known concentrations. However, this protocol does not mimic physiological conditions accurately, where compounds are routinely added from a solvent vehicle, such as DMSO or ethanol, to the cell medium or administered to the animals. Therefore, addition of drugs to the membrane-bathing solution in channel reconstitution experiments seems to be a more justified protocol. In this case, an independent method allowing for verification of the compound’s partitioning into the lipid membrane is required (see Part 5) or, otherwise, strong statements on the lack of interaction with VDAC should be avoided.

## Screening Non-Polar Compounds for Vdac Functional Control

VDAC’s unique physiological role as a mitochondrial regulator (Lemasters and Holmuhamedov, 2006) makes it a strong candidate for pharmaceutical intervention in diseases such as cancer, cardiovascular disorders, diabetes, and neurodegeneration where mitochondrial dysfunction is known. Proteomics and other recently developed experimental approaches identified a plethora of different pharmacological agents targeting VDAC, but rationalization of their molecular mechanisms of action is mostly absent or incomplete. In many cases, the effect on VDAC function is unclear and a bona fide binding is not fully validated. In this context, it is crucial to establish reliable experimental and interpretational strategies to determine unambiguously a compound interaction with VDAC and its consequences on channel function and on mitochondrial physiology. This is the first necessary step in establishing pharmacologically relevant candidates for therapeutic applications based on VDAC-targeting.

In this vein, an impressive number of compounds have been reported to target VDAC and consequently affect its channel function. An exhaustive recent review of the compounds thought to affect VDAC function for use in anti-cancer therapy has been compiled by Reina and De Pinto (Reina and De Pinto, 2017). The authors make an essential conclusion that there is neither a common structural motif among found anti-cancer drugs acting on VDAC, nor a well-defined specific binding site or catalytic cleft in VDAC protein. They conclude that VDAC, from a traditional point of view, “is not a druggable target protein” (Reina and De Pinto, 2017). This situation obviously impedes an intelligent design of drugs targeting VDAC and creates difficulties in interpretation of the effects of drugs on VDAC’s function and, consequently, on mitochondrial physiology.

Most of the low-molecular weight drugs found by the high-throughput approaches to bind to VDAC are non-polar or hydrophobic compounds (Reina and De Pinto, 2017). This is understandable, considering that an utmost requirement for the drugs is to pass the cell membrane barrier. Therefore, in this review, we restrict our discussion to mostly non-polar compounds which were found to interact with VDAC using recent technological advances in proteomic, biochemical, and computational methods (Table 1). In particular, here we focus on comparing the effects of four non-polar compounds, cholesterol, allopregnanolone, olesoxime, and propofol, on the VDAC channel properties. Each of these four compounds have been shown by different biochemical methods to bind to VDAC, and their effect on channel function and bilayer properties has been tested. We also review other hydrophobic compounds that have been demonstrated to interact with VDAC, but comprehensive functional characterization is not available yet.

**TABLE 1:** Identified VDAC-binding hydrophobic compounds.

Compound	Method of VDAC binding determination	Effect on VDAC gating	Effect on VDAC conductance	Effect on bilayer properties at working concentrations
Cholesterol 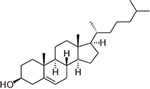	Photoaffinity labeling (Budelier et al., 2017) MD Simulations (Weiser et al., 2014b)	No (Queralt-Martin et al., 2019)	No (Queralt-Martin et al., 2019)	Yes (van Blitterswijk et al., 1987; Weinrich et al., 2009)
Allopregnanolone 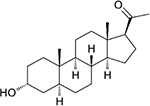	Photoaffinity labeling (Cheng et al., 2019)	No (Cheng et al., 2019)	No (Cheng et al., 2019)	Yes (Alakoskela et al., 2007; Cheng et al., 2019)
Olesoxime 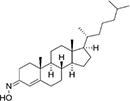	Photoaffinity labeling (Bordet et al., 2007)	Yes (Rovini et al., 2019)	No (Rovini et al., 2019)	No (Rovini et al., 2019)
Propofol 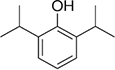	Photoaffinity labeling (Weiser et al., 2013; Weiser et al., 2014a)	Yes (Weiser et al., 2014a)	No (Weiser et al., 2014a)	No (Bahri et al., 2007; Weiser et al., 2014a)
Itraconazole 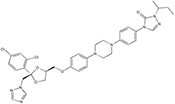	Photoaffinity labeling and affinity pulldown (Head et al., 2015)	TBD	TBD	TBD
Efsevin 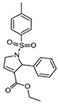	Affinity pulldown (Shimizu et al., 2015)	Yes (Wilting et al., 2020)	No (Wilting et al., 2020)	TBD
Erastin 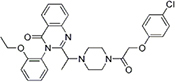	Binds to VDAC2; Affinity pulldown (Yagoda et al., 2007)	No (Maldonado et al., 2013)	No (Maldonado et al., 2013) Increases VDAC2 permeability to NADH (Bauer et al., 2011)	Yes (Jacobs, personal communication)
Sulindac sulfone 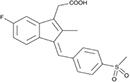	Affinity pulldown (Aono et al., 2018)	TBD	TBD	Yes (Fan and Shen, 1981; Santos et al., 2008)
WEHI-9625 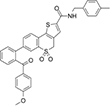	Photoaffinity labeling (van Delft et al., 2019)	TBD	TBD	TBD

**Cholesterol** is an intrinsic component of most mammalian cell membranes (van Blitterswijk et al., 1987; van Meer et al., 2008) found in high concentrations, up to 8%, within the MOM compared to 2% in the IM (de Kroon et al., 1997; Flis and Daum, 2013). It is also a precursor to many important hormones which are synthesized in mitochondria (Cheng and Kimura, 1983; Ardail et al., 1990). Cholesterol is well-known as a membrane-modifying agent, with the ability to bind and modify membrane proteins (Hanson et al., 2008; Weinrich et al., 2009). Cholesterol was first shown to purify out with VDAC in 1989 (De Pinto et al., 1989), with more recent studies confirming VDAC-cholesterol association using photolabeling coupled to mass spectrometry and MD simulations (Hulce et al., 2013; Weiser et al., 2014b). All three mammalian VDAC isoforms have been identified as proteins interacting with cholesterol (Hulce et al., 2013). NMR studies (Hiller et al., 2008) have shown multiple cholesterol binding sites in murine VDAC1 (mVDAC1) that were later confirmed by MD simulations (Weiser et al., 2014b). An additional characterization using photoaffinity labeling with mass spectrometry identified a cholesterol binding pocket in the lipid-facing outer surface of the mVDAC1 β-barrel coordinated by the membrane-facing residue E73 (Budelier et al., 2017). Cholesterol is, maybe, the clearest example of a hydrophobic compound with a well-established interaction with VDAC that, however, does not affect its basic channel properties, such as open-channel conductance or voltage-induced gating (Table 1 and Figure 4A).

The lack of a significant effect of cholesterol on VDAC channel properties was first demonstrated for plant VDAC isolated from *P. coccineus* (Mlayeh et al., 2010), where it was shown that cholesterol and phytosterols only slightly modulate the plant VDAC ion selectivity, with stigmasterol altering its voltage-gating. The differentiated effect of mammalian cholesterol and phytosterols on plant VDAC could be understood as a specific affinity between plant VDAC and certain phytosterols (Mlayeh et al., 2010). However, lately the lack of effect of cholesterol on the VDAC single-channel conductance and voltage-gating properties was confirmed on recombinant mVDAC1 (Queralt-Martin et al., 2019) and on VDAC purified from rat liver mitochondria (Figure 4A; Rovini et al., 2019). A plausible explanation was found in experiments with mVDAC1 E73Q and E73A mutants where it was demonstrated that the main residue in the cholesterol binding pocket, E73, appeared not to be involved in the gating process (Queralt-Martin et al., 2019).

**Allopregnanolone** is an endogenous neurosteroid derivative of the reproductive hormone progesterone, known to allosterically affect the GABA-A receptor (Akk et al., 2009; Reddy, 2010). It is also a natural anesthetic thought to play a role in fetal behavior (Korneyev and Costa, 1996; Mellor and Diesch, 2006). More recently, allopregnanolone has been approved as a treatment for postpartum depression (FDA approval)^1^. Photoaffinity labeling coupled with mass spectrometry has shown that allopregnanolone interacts with mVDAC1 and competes with cholesterol for the same binding pocket (Cheng et al., 2019). Not surprisingly, given the lack of cholesterol effect on VDAC gating (Queralt-Martin et al., 2019; Rovini et al., 2019), consecutive allopregnanolone additions to both sides of the membrane, where multiple VDAC channels were inserted, yielded no effect on VDAC gating properties (Figure 4B; Cheng et al., 2019). Therefore, allopregnanolone is another example of a non-polar compound that was found to bind VDAC using biochemical methods, but not to affect its basic channel properties, conductance and gating.

**FIGURE 6:**
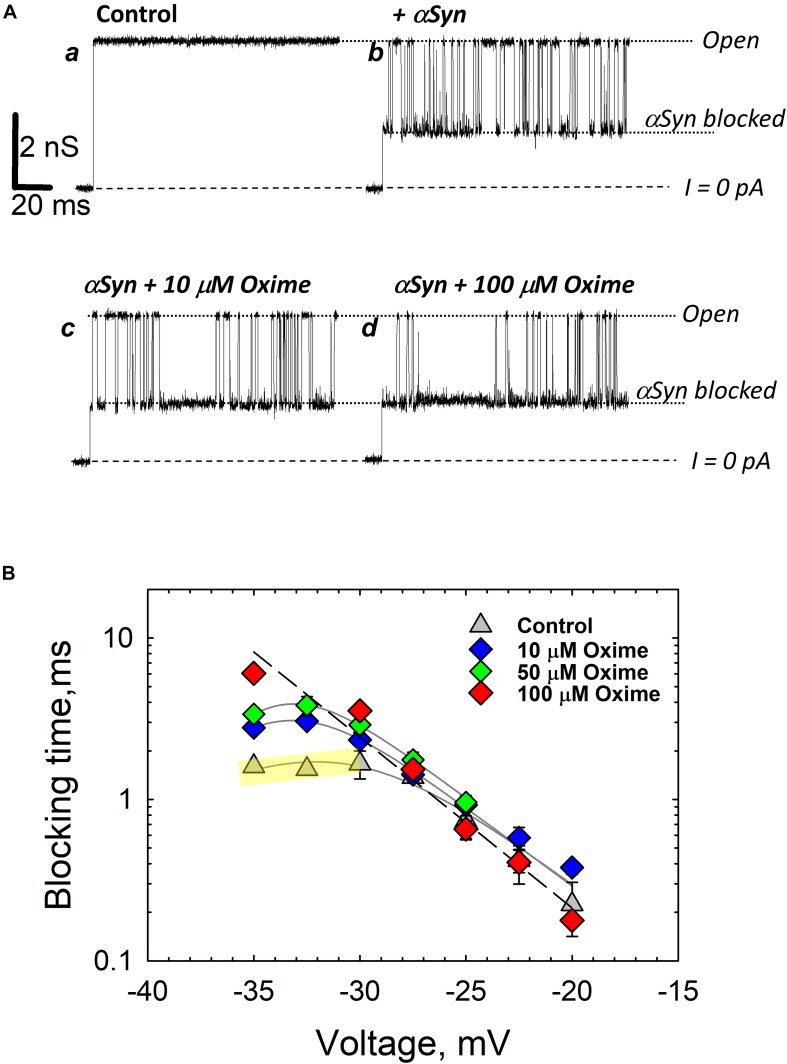
Olesoxime prevents α-synuclein translocation through reconstituted VDAC. **(A)** Records of ion current of the same single VDAC channel reconstituted into a planar lipid bilayer formed from a DOPC/DOPE (1:4 w/w) mixture with 5% (w/w) of cholesterol before (*trace a*) and after addition of 50 nM of α-synuclein to the *cis* compartment (*trace b*). Traces *c* and *d* were obtained after consequent additions of olesoxime to the final concentrations of 10 and 100 μM in the membrane-bathing solution containing 1 M KCl buffered with 5 mM HEPES at pH 7.4. All records were taken at –30 mV applied voltage. Dotted lines indicate VDAC open and α-synuclein-blocked states and dashed lines show zero current. **(B)** The voltage dependences of mean blocking times of α-synuclein-induced blockages obtained at different olesoxime concentrations on the same channel. The regime of α-synuclein translocation through VDAC, corresponding to a decrease of blockage time with voltage amplitude, is highlighted in yellow for the data obtained in control conditions as illustrated by trace *b* in **(A)**. At | V| > 27.5 mV the blockage time increases with olesoxime concentration. The voltage dependence of the mean blockage time starts deviating from the translocation regime, indicating inhibition of α-synuclein translocation through the channel. Figure adapted by permission from Springer Nature: Springer International Publishing Cellular and Molecular Life Sciences (Rovini et al., 2019), Copyright (2019).

**Olesoxime**, also known as TRO19622, is a mitochondria-targeting experimental synthetic neuroprotective compound derived from cholesterol (Bordet et al., 2007). Olesoxime generated considerable interest for its ability to protect neurons and mice from a wide range of neurodegenerative conditions including spinal muscular atrophy, Parkinson’s and Huntington’s diseases, Huntington disease, amyotrophic lateral sclerosis, and chemotherapy side effects (Bordet et al., 2007; Martin, 2010; Rovini et al., 2010; Gouarne et al., 2015). However, clinical trials of olesoxime have had mixed results, leading in termination of the last ongoing trial^2^. The molecular mechanism of olesoxime protection is not well understood [for review see Weber et al. (2019)]. It was shown that olesoxime accumulates in mitochondrial membranes and a photolabeling study identified VDAC together with another major MOM protein –TSPO, the mitochondrial cholesterol transporter – as one of the MOM targets for olesoxime (Bordet et al., 2007; Bordet et al., 2010). Because olesoxime is a cholesterol-like molecule, it was not expected to affect reconstituted VDAC channel properties. Instead, it was found (Rovini et al., 2019) that olesoxime promotes VDAC gating (Figure 4C), though it does not impact VDAC open channel conductance (Figure 6A). Addition of 20% olesoxime to the membrane-forming lipid mixture increases VDAC gating by decreasing the minimum conductance (*G*_*min*_). Olesoxime was added to the membrane-forming lipid mixture to keep the same protocol as in experiments with cholesterol and to make an adequate comparison between the effects of two sterols on channel properties. Therefore, olesoxime is an example of a hydrophobic compound that has been shown to bind to VDAC *and* to affect channel voltage-gating properties.

Olesoxime has another potentially important effect on VDAC by modulating its interaction with a cytosolic protein, α-synuclein. α-Synuclein is a natively unfolded neuronal protein intimately associated with Parkinson’s disease (Kim et al., 2014; Goedert et al., 2017). It is well established that α-synuclein is associated with mitochondria, causing impairment of the mitochondrial respiratory complexes (Elkon et al., 2002; Devi et al., 2008; Ludtmann et al., 2018), oxidative stress (Hsu et al., 2000), and fission (Chinta et al., 2010; Nakamura et al., 2011). However, how exactly α-synuclein is involved in neurodegeneration and how it enters mitochondria to target electron transport complexes in the IM remains mainly unknown. Recently, it was found that VDAC could be one of the pathways for α-synuclein translocation across the MOM (Rostovtseva et al., 2015) and that olesoxime, by interacting with VDAC, hinders α-synuclein translocation through the channel (Rovini et al., 2019) (type 3 interaction in Figure 5). Using VDAC single-channel experiments it was shown that olesoxime modifies the characteristic times of α-synuclein-induced VDAC blockages (Figure 6). Based on kinetic analysis, it was suggested that olesoxime fulfills its action by affecting α-synuclein translocation through, and most likely binding to, VDAC. In the proposed model (Figure 5), olesoxime interacts with the hydrophobic exterior of the VDAC’s β-barrel, targeting it from the lipid medium (type 1 interaction). Notably, under this mechanism, hydrophobic olesoxime does not interact with the hydrophilic VDAC pore which means that channel open state conductance does not change in the presence of olesoxime (Figure 6A, compare the open state conductance in the control trace *a* and in the presence of olesoxime, traces *c* and *d*). The same is true for cholesterol – it does not affect VDAC open state conductance (Queralt-Martin et al., 2019).

Taking together all above relationships between olesoxime, VDAC, and α-synuclein, we can conclude that olesoxime is an example of a hydrophobic drug that binds to VDAC, affects its gating properties but, what seems to be more physiologically important, interferes with VDAC interaction with its cytosolic regulator, α-synuclein. This is a novel mechanism, where a neuroprotective compound inhibits transport of a neurodegenerative-related protein into mitochondria through interaction with another mitochondrial membrane protein (Rovini et al., 2019).

**Propofol** is a general anesthetic thought to act as GABA receptor antagonist (Yip et al., 2013). Propofol was found to bind to VDAC *in vivo* in *Xenopus laevis* tadpoles and in rat brains through photoaffinity labeling coupled with mass-spectrometry (Weiser et al., 2013, 2014a). Two isoform-conserved VDAC residues were found as propofol binding sites, G56 and V84 (Weiser et al., 2014a). In experiments with reconstituted VDAC, propofol was demonstrated to increase VDAC voltage-gating manifested as ∼20% decrease of *G*_*min*_ at negative potentials in the normalized *G/V* plot (Figure 4D; Weiser et al., 2014a). At the same time, propofol does not affect VDAC open-channel conductance. Propofol, along with olesoxime, is another example of a hydrophobic compound that binds to VDAC *and* affects its voltage-gating properties.

**Itraconazole** is a widely used anti-fungal agent, which is now recognized as a potent anti-angiogenic drug (Chong et al., 2007). It was found to inhibit the target of rapamycin (mTOR) signaling, a pathway that integrates nutritional signals and directs cellular growth (Head et al., 2017). VDAC1 was identified as the major itraconazole-binding protein through a combination of affinity pulldown coupled to mass spectrometry and itraconazole photoaffinity labeling (Head et al., 2015). It was shown that VDAC1 knockdown suppressed mTOR activity and cell proliferation in human umbilical vein cells and made cells resistant to AMPK activation and mTOR inhibition by itraconazole. A decrease in cellular ATP levels along with increased mitochondrial permeability to calcium observed upon itraconazole treatment together with itraconazole direct binding to VDAC1 let authors to speculate that these effects are consistent with inhibition of VDAC permeability for ATP by itraconazole (Head et al., 2015). However, without direct evidence of itraconazole promoting the VDAC closed state which restricts ATP transport through the pore (Rostovtseva and Colombini, 1996), a proposed model of VDAC1 inhibition by itraconazole mediating activation of AMPK remains only plausible speculation.

**Efsevin** is a novel antiarrhythmic compound shown to alter calcium signaling in zebrafish. Efsevin was demonstrated to bind to zebra fish VDAC2 (zfVDAC2) by an affinity pulldown assay (Shimizu et al., 2015). Efsevin is thought to enhance mitochondrial uptake of calcium through VDAC2, thereby preventing calcium accumulation in the cytosol (Schweitzer et al., 2017). Lately, using an *in vitro* system of reconstituted zfVDAC2 channels, Wilting et al. (2020) found that efsevin promotes channel closure and shifts its selectivity toward less anionic thus, according to the previous studies (Rostovtseva et al., 2005; Tan and Colombini, 2007), ultimately causing an increase of VDAC2 permeability for calcium. Authors concluded that this mechanism could explain the efsevin-induced facilitated transfer of calcium from the sarcoplasmic reticulum into mitochondria in zebra fish. Interestingly, in these studies, efsevin has not been observed to be involved in apoptosis (Schweitzer et al., 2017; Wilting et al., 2020) despite the accepted role of VDAC2 in apoptosis through recruitment of Bax or Bak (Cheng et al., 2003; Chin et al., 2018).

**Erastin** is an anti-cancer drug known to induce ferroptosis, a distinct form of cell death, dependent on lipid peroxidation and iron accumulation (Dixon et al., 2012). An early study on erastin-induced ferroptosis recognized VDAC as necessary for erastin function, with erastin found to directly bind to human VDAC2 and VDAC3 using affinity pulldown coupled with mass spectrometry (Yagoda et al., 2007). It has been reported that erastin decreases the rate of NADH oxidation in isolated yeast mitochondria expressing a single mouse VDAC isoform (Yagoda et al., 2007). The authors interpreted their results suggesting that erastin affects VDAC gating, possibly switching its ion selectivity and allowing cationic species into mitochondria. In the follow up work, Stockwell and coauthors using an elegant *in vitro* liposome assay showed an opposite effect of erastin: a ∼50% increase of permeability to NADH of hVDAC2 doped liposomes (Bauer et al., 2011). Although the authors do not speculate on the detailed mechanism of erastin-mediated cell death, they suggest that erastin acts through VDAC by modulating metabolite flux. Later, in cell and reconstitution studies, erastin was found to block and reverse mitochondrial depolarization induced by microtubule destabilizers in intact HepG2 cells and antagonize tubulin-induced VDAC blockage in planar bilayers (Maldonado et al., 2013). In this study erastin was found to modulate VDAC permeability indirectly, by disrupting the interaction between VDAC and dimeric tubulin, another known potent VDAC regulator (Rostovtseva et al., 2008b; Gurnev et al., 2011; Maldonado et al., 2011). Dimeric tubulin, like α-synuclein, induces reversable blockages of VDAC conductance (Rostovtseva and Bezrukov, 2008). Hence, Maldonado and coauthors suggested an entirely different mechanism of erastin anti-cancer effect where erastin prevents and reverses tubulin-induced VDAC blockage to promote mitochondrial metabolism and antagonize Warburg metabolism in cancer cells (Maldonado et al., 2013; Maldonado and Lemasters, 2014). Contrary to the previous work (Bauer et al., 2011), Maldonado and coauthors (Maldonado et al., 2013) reported that erastin has no effect on VDAC conductance when added alone in the absence of tubulin. Therefore, erastin is an example of a drug that binds to VDAC and *does not* affect its basic channel properties but does modify VDAC’s interaction with its cytosolic protein partners, similar to the effect of olesoxime on α-synuclein-VDAC interaction described above.

**Sulfone-containing compounds,** synthetic WEHI-9625 and metabolite of anti-inflammatory drug sulindac, sulindac sulfone, were recently found to bind to VDAC. **WEHI-9625** is a recently identified tricyclic sulfone-containing low molecular weight compound which binds to VDAC2 and promotes its ability to inhibit BAK-induced apoptosis (van Delft et al., 2019). Importantly, WEHI-9625 blocks apoptosis upstream of mitochondrial damage and, therefore, BAK activation can be blocked with a small molecule targeting VDAC2. Using photocrosslinking experiments in conjugation to biotin coupled mass spectrometry, it was found that WEHI-9625 directly binds to VDAC1 and VDAC2, but less well to VDAC3. However, VDAC isoform deletion experiments demonstrated that only VDAC2, among the three isoforms, is essential for WEHI-9625 and its analogs to block BAK-mediated apoptosis. Consequently, WEHI-9625, along with olesoxime and erastin, achieve their anti-cancer or anti-apoptotic action by interfering with VDAC interaction with its cytosolic partners, BAK, α-synuclein, or tubulin. **Sulindac Sulfone** is an anti-cancer metabolite of the non-steroidal anti-inflammatory drug sulindac (Thompson et al., 1997). Similar to itraconazole, sulindac sulfone was shown to inhibit mTOR signaling, dependent on its interaction with VDAC. Using affinity pulldown coupled with mass spectrometry, it was demonstrated that sulindac sulfone binds to VDAC1 and VDAC2 (Aono et al., 2018). The presence of sulfone groups on WEHI-9625 and on related antiapoptotic compounds as well as sulindac sulfone (Table 1) suggests that the sulfone moiety may be important for VDAC binding, thus opening a new promising venue for the VDAC-targeting drugs design. However, the effect of sulfonic compounds on VDAC channel properties has not been tested yet, leaving the mechanism of their interaction with VDAC unknown.

## Probing Lipid Bilayer Properties With Gramicidin a Channel

**FIGURE 7:**
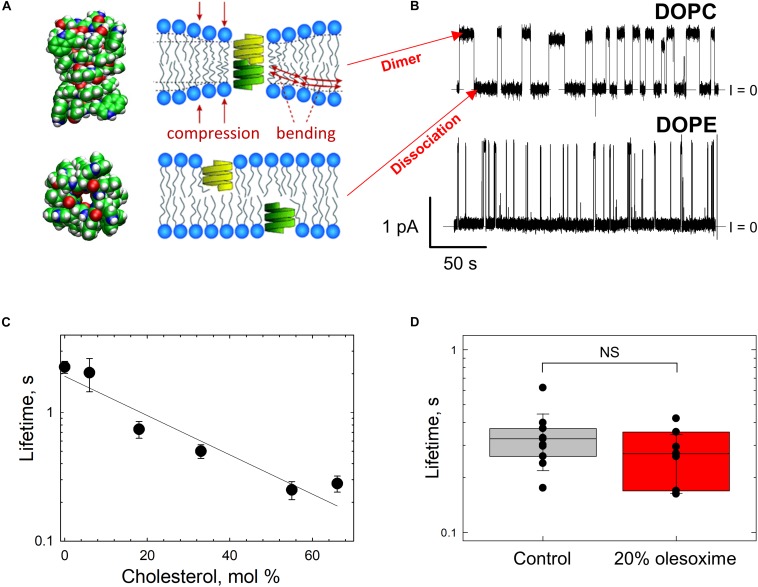
Gramicidin A channel lifetime reports on lipid bilayer mechanics. **(A)** An atomic model of a gA conducting dimer created from the PDB file 1JNO (left panel). Monomers of gA have to deform the bilayer to create a conducting dimer. Membrane deformation energy depends on the lipid packing stress and membrane thickness (right panel), modified from Lundbaek et al. (2010). Copyright 2010 National Academy of Sciences. **(B)** Representative current traces of gA channels obtained in a bilayer formed from DOPC (upper trace) and DOPE (bottom trace) membranes in 1 M KCl solutions buffered with 5 mM HEPES at pH 7.4. The gA channel lifetime in DOPC membranes decreases with addition of DOPE. The applied voltage was 50 mV. Current records were filtered using an averaging time of 30 ms. Dashed lines indicate the zero-current level. Modified from Rostovtseva et al. (2008a). Copyright (2008), with permission from Elsevier. **(C)** The effect of addition of cholesterol to DOPC bilayers on the gA channel lifetime. Cholesterol content is given as its concentration in the lipid mixture used for bilayer formation. Membranes were bathed in 0.1 M KCl aqueous solution buffered at pH 7.2. The applied voltage was 100 mV. Adapted with permission from Weinrich et al. (2009). Copyright 2009 American Chemical Society. **(D)** Channel lifetimes in DOPE/DOPC (4:1) bilayers without and with 20% (w/w) of olesoxime. Olesoxime does not affect the channel lifetime. NS (not significant): *p* ≥ 0.05. The membrane-bathing solutions contained 1 M KCl buffered with 5 mM HEPES. Adapted by permission from Springer Nature: Springer International Publishing Cellular and Molecular Life Sciences (Rovini et al., 2019), Copyright (2019).

While there is a variety of biophysical and biochemical methods available to probe lipid bilayer physical properties, the approach of using the channel-forming polypeptide gramicidin A (gA) is advantageous, because it allows testing exactly the same system of planar lipid membranes as in VDAC reconstitution experiments. The gA channel is well-known as a highly sensitive molecular probe of planar bilayer mechanics (Lundbaek and Andersen, 1999; Suchyna et al., 2004; Lundbaek et al., 2005; Rostovtseva et al., 2006; Weinrich et al., 2009). This channel has a unique structure – the right-handed β6,3-helical dimer known to better than 1Å resolution (Figure 7A; Townsley et al., 2001). Formation of the conducting dimer of ∼2.2 nm length (Elliott et al., 1983) from two opposed gA monomers requires a local bending of the bilayer (Figure 7A, right). This creates a dissociative force acting on the conducting dimer that decreases its lifetime, the characteristic time that an individual conducting dimer exists before dissociating into non-conducting monomers (Figures 7A,B). Consequently, gA lifetime was shown to be extremely sensitive to the bilayer thickness, lipid packing stress, and several other factors (Lundbaek and Andersen, 1999; Bezrukov, 2000; Rostovtseva et al., 2008a) [for an in-depth review see Lundbaek et al. (2010)]. For example, gA channels are distinctly different when incorporated into the membranes made of lamellar (DOPC) and non-lamellar (DOPE) lipids (Figure 7B). The channel lifetime is ∼10-fold longer in DOPC membranes than in pure DOPE membranes (Rostovtseva et al., 2008a; Figure 7B). The hydrophobic thickness of DOPE bilayers is larger than that of DOPC by ∼0.3 nm (Gawrisch et al., 1992; Nagle and Tristram-Nagle, 2000), which leads to a significant increase in the mismatch between the length of the gA dimer and bilayer thickness. This increases the disjoining forces acting on the conducting dimer, thus promoting its dissociation and decreasing its lifetime (Rostovtseva et al., 2008a).

It was established that different amphiphiles could affect gA channel parameters indirectly through the membrane surrounding the channel by changing the lipid bilayer properties (Lundbaek et al., 2010). Therefore, in addition to providing information on the effects of non-polar compounds on bilayer properties revealed by gA, this channel serves as a sensitive indicator of compound’s partitioning to the membrane.

The results of gA experiments with cholesterol and olesoxime illustrate how such experiments help to resolve the issue of membrane partitioning. For instance, cholesterol added to the membrane-forming lipid solution causes a decrease in gA channel lifetime (Figure 7C; Weinrich et al., 2009). Addition of 55 mol% cholesterol to DOPC membrane causes a ∼10 times reduction of gA lifetime (Figure 7C) due to the increase in the lipid packing stress or so called membrane stiffness (Lundbaek et al., 1996). In contrast, the synthetic steroid olesoxime which affects VDAC gating (Figure 4C), does not change gA lifetime measurably when added to the membrane up to 20 mol% (Figure 7D; Rovini et al., 2019). This result allows us to conclude that olesoxime most likely affects VDAC gating by binding directly to its β-barrel exterior surface at the protein-lipid interface.

## Conclusion

Pharmacological targeting of VDAC has a significant therapeutic potential in several diseases including cancer, neurodegeneration, cardiovascular, and obesity where altering mitochondrial physiology through VDAC may be beneficial. Moreover, this could be a useful approach to manipulate mitochondrial function in cell and animal models in basic research. In this context, non-polar compounds represent a promising avenue of research because of their ability to partition into the MOM and modulate VDAC function via the lipid bilayer. Compounds can affect its voltage gating by directly interacting with VDAC at the protein-lipid interface or indirectly, through the “lipid matrix effect” by modifying the properties of the lipid bilayer surrounding the channel. By modulating gating, compounds promote channel open or closed states, consequently leading to the increase or decrease of ATP and other metabolites fluxes through VDAC, thus affecting mitochondrial bioenergetics. In recent years VDAC has been recognized as a docking site for cytosolic proteins to modulate mitochondrial function. In this paradigm, compounds that bind to VDAC may not affect its intrinsic channel properties such as gating, conductance, or selectivity, but instead exert their function by modulating VDAC interaction with its partner proteins. Indeed, the drugs olesoxime and WEHI-9625 have been shown to affect mitochondrial physiology by disrupting VDACs interaction with α-synuclein and Bak, respectively. These compounds, one derived from cholesterol and the other being a tricyclic ring with a sulfone moiety, may represent structures from which new VDAC-targeting compounds can be developed. Altogether, the available data with reconstituted VDAC allow us to conclude that observation of binding of a compound of interest to VDAC does not necessarily mean that it would affect its basic channel characteristics. However, the compound may modify other physiological aspects of VDAC functioning through altering its interaction with cytosolic partner proteins or formation of complexes with other mitochondrial membrane proteins. The complementary testing is imperative to determine the functional implications of VDAC targeting by hydrophobic compounds.

## Author Contributions

All authors listed have made a substantial, direct and intellectual contribution to the work, and approved it for publication.

## Conflict of Interest

The authors declare that the research was conducted in the absence of any commercial or financial relationships that could be construed as a potential conflict of interest.

## Footnotes

1 https://www.accessdata.fda.gov/drugsatfda_docs/nda/2019/211371Orig1s000MultidisciplineR.pdf)

2 https://clinicaltrials.gov/ct2/show/NCT02628743
